# Short-Term 8-Foot up and Go Responsiveness in Institutionalized vs. Community-Dwelling Older Adults

**DOI:** 10.3390/sports14020047

**Published:** 2026-02-02

**Authors:** Filipe Rodrigues, Diogo Monteiro, Pedro Forte, António Miguel Monteiro

**Affiliations:** 1 ESECS—Polytechnic of Leiria, 2411-901 Leiria, Portugal; diogo.monteiro@ipleiria.pt; 2 Research Center in Sport, Health and Human Development (CIDESD), 5000-558 Vila Real, Portugal; 3 Department of Sports Sciences, Instituto Politécnico de Bragança, 5300-253 Bragança, Portugal; pedromiguel.forte@iscedouro.pt (P.F.); mmonteiro@ipb.pt (A.M.M.); 4 Research Centre for Active Living and Wellbeing (LiveWell), Instituto Politécnico de Bragança, 5300-253 Bragança, Portugal; 5 Department of Sports, Higher Institute of Educational Sciences of the Douro, 4560-708 Penafiel, Portugal

**Keywords:** aging, fall risk, body mass index, institutionalization, functional mobility, sarcopenia, body composition

## Abstract

This study aimed to examine the combined effects of Body Mass Index (BMI) and living setting on fall risk trajectories in older adults over a 16-week period. A total of 124 older adults were recruited from nursing homes (*n* = 65; M_age_: 84.4 ± 7.7 years) and community settings (*n* = 59; M_age_: 70.3 ± 3.6 years). Participants were stratified by BMI (normal weight, overweight, and obesity) and assessed for functional mobility using the 8-foot Timed Up and Go test at baseline and after 16 weeks. A 3 × 2 × 2 repeated-measures GLM using the multivariate approach (Pillai’s Trace) revealed significant main effects for time (*p* < 0.001) and Living Setting (*p* < 0.001), but not for BMI (*p* = 0.408). A significant time × living setting interaction (*p* < 0.001) indicated that institutionalized older adults demonstrated a significant reduction in 8-foot Up-and-Go completion time (~16%), whereas community-dwelling peers maintained their baseline levels. These findings suggest that the observed reduction in time reflects a test familiarization effect rather than physiological improvement. Analysis revealed that the living setting profile was strongly associated with performance, showing a more significant link to functional decline than BMI-defined weight status, as no significant differences were found across BMI categories.

## 1. Introduction

Current demographic shifts indicate a substantial increase in the older adult population, presenting structural challenges for public health systems regarding the preservation of quality of life and autonomy. With advancing age, a progressive decline in neuromuscular function and physical fitness is observed, often exacerbated by sedentary lifestyles [1,2]. This decline frequently progresses to sarcopenia, a syndrome characterized by the progressive and generalized loss of muscle strength and mass, which is intrinsically linked to physical frailty and an increased risk of adverse events, such as falls [3]. The loss of muscle strength does not act in isolation as it interacts dynamically with changes in body composition, namely increased adiposity, creating high-risk phenotypes such as sarcopenic obesity, where excess weight coexists with functional deficit [4].

Fall prevention is, therefore, a clinical priority, as falls represent one of the leading causes of morbidity and loss of independence in older adults. To monitor fall risk and functional mobility, the timed up and go test and its adapted versions, specifically the 8-foot Up-and-Go (8UG), have established themselves as the gold standard in geriatric assessment due to their simplicity, speed of execution, and high correlation with dynamic balance and functional gait [5,6]. Crucially, the 8UG also places demands on executive function and processing speed, making it a sensitive composite measure of motor, and potentially cognitive decline. The validity of the 8UG has been extensively tested, not only in comparison with complex test batteries but also recently validated against advanced optoelectronic systems, highlighting its robustness in quantifying balance variables in adults over 65 years of age [7]. Furthermore, the intra- and inter-rater reliability of the 8UG has been specifically confirmed in the Portuguese older adult population, reinforcing its utility as a monitoring measure in national community and institutional contexts [8].

Scientific literature has sought to establish normative values for the 8UG and other functional fitness tests, allowing clinicians to situate an individual’s performance relative to their peers. Since the seminal works of Rikli and Jones in the USA [9], followed by descriptive meta-analyses that established mean reference times by age groups [10], several studies have expanded these norms globally. Robust data now exist for populations in Canada [11], Spain [12], and more recently in Asian regions such as Thailand [13], Hong Kong [14], and Taiwan [15], as well as in rural populations in Malaysia, where performance was also stratified by cognitive status [16]. These cross-cultural comparisons are vital, as recent studies indicate that sociodemographic factors and regional lifestyles significantly influence functional fitness [17]. For instance, recent systematic reviews [18] and normative studies in South America, namely in Chile [19], suggest that patterns of functional decline may vary according to socioeconomic context.

In the specific context of Portugal, research has been prolific in characterizing functional fitness. Large-scale studies in Madeira [20] and on the mainland [21] have demonstrated that Portuguese older adults tend to present a marked functional decline with age, sometimes greater than that observed in North American peers, placing them at higher risk of losing independence. Longitudinal investigations, comparing cohorts from 2008 and 2018, warn of a concerning trend of decreased physical fitness, especially in aerobic endurance, in successive cohorts of Portuguese older adults [22]. Studies focused on the Leiria community corroborate this need for continuous monitoring, evidencing fitness levels that require intervention [23]. Furthermore, studies in low-income communities, such as in Brazil, reinforce that social vulnerability amplifies functional decline, necessitating accessible assessment strategies like the 8UG [24].

The relationship between 8UG performance and the risk of mortality or hospitalization or institutionalization is another area of intense investigation. Elevated 8UG times have been shown to be independent predictors of all-cause mortality in population cohorts, as observed in Peru [25]. In a hospital setting, a cut-off point of 12 s for the standard 3 m TUG was recently proposed to identify older adults at risk of hospitalization-associated functional decline [26]. However, 8UG performance does not depend solely on motor capacity as it is also influenced by psychological factors such as well-being and self-efficacy [27], as well as the fear of falling, which can alter motor strategies even in older adults with no history of falls [28]. Complexity increases when considering cognitive demands; 8UG performance under dual-task conditions reveals deficits that the simple test may not detect, especially in older adults with a history of falls [29].

One of the most critical factors influencing physical performance is body composition, specifically BMI, although its predictive value in distinct environmental contexts remains under scrutiny. Literature points to a non-linear relationship, sometimes U-shaped, where both low weight and obesity are associated with worse functional performance [30]. However, recent studies suggest that the “obesity penalty” may not be universal. While excess fat mass imposes a mechanical overload that limits mobility in community settings, lower BMI in geriatrics is often a proxy for sarcopenia and frailty, potentially carrying a higher risk of functional decline than overweight status in vulnerable populations [31,32]. In cases of morbid obesity (i.e., Class III), changes in gait and function are profound and distinct from those observed in simple overweight [31]. The condition of “sarcopenic obesity” is particularly dangerous as older adults with this condition exhibit worse static and proactive balance compared to those with obesity alone or sarcopenia alone, as demonstrated in recent analytical studies [32]. Moreover, muscle quality, not just quantity, appears to be determinant since in obese older women, the muscle quality index was a significant predictor of 8UG performance [33].

However, the relationship between BMI and fall risk is not without controversy. Some data suggest that central (abdominal) obesity may be a stronger predictor of falls than general obesity defined by BMI [34]. Interestingly, in older adults participating in public physical activity programs, weak correlations were found between the 8UG and anthropometric indicators, suggesting that exercise may mitigate the negative effects of excess weight [35]. This view is supported by recent studies indicating that muscle strength is a more potent mediator of fall risk than BMI per se. That is, muscle strength “matters more” than body weight in fall prevention [36]. Beyond individual biological factors, the living setting (community-dwelling vs. institutionalized) is a critical determinant of the older adult’s functional trajectory. Literature consistently suggests that institutionalized older adults exhibit lower physical fitness levels and more rapid functional decline compared to their community-dwelling peers, driven by reduced motor stimuli and sedentary behaviors [8]. Crucially, the institutional environment may alter the relationship between anthropometry and mobility. The obesity paradox, where overweight individuals exhibit better survival or functional outcomes than those with normal or low weight, has been documented in chronic conditions but rarely explored in nursing homes regarding fall risk [33]. It is plausible that in institutionalized settings, the lack of physical stimulus makes normal weight individuals more susceptible to sarcopenia, whereas higher adiposity might offer a metabolic or mechanical reserve, limiting the usefulness of BMI as an isolated predictor. For instance, sarcopenic obesity might progress more rapidly in nursing home settings due to forced inactivity, a scenario that the 8UG test could detect early.

Although the literature is extensive, specific gaps justify further investigation. Many existing studies are cross-sectional, offering only a momentary snapshot of functional status [37,38]. There is a lack of studies analyzing the stability or variation of fall risk over the short term as a function of distinct BMI categories, using validated cut-off points for physical independence, such as those proposed for the Portuguese [39] and Chinese [40] populations. Understanding whether older adults with obesity, overweight, and normal weight respond differently to the passage of time, even in a short interval, is crucial for designing personalized intervention strategies. Additionally, most existing studies focus either exclusively on community-dwelling older adults [20,21] or solely on institutionalized populations, rarely comparing the two directly in longitudinal designs. To the best of our knowledge, no study within the analyzed literature has investigated the interaction between BMI categories and living settings. It remains to be determined whether the impact of obesity on fall risk is uniform across settings or if the institutional environment acts as a catalyst for faster functional decline in overweight individuals [41]. Understanding this interaction is vital, as it could shift fall prevention guidelines from a one-size-fits-all approach to one stratified by both body composition and environmental context.

The present study aims to examine the independent and combined effects of body composition and environment by comparing fall risk (assessed by 8UG performance) across three BMI categories and two Living Settings (Community vs. Institution) over a period of 16 weeks. We acknowledge that these settings represent two distinct cohorts with different biological profiles (e.g., age and frailty levels). Thus, the study analyzes how these distinct profiles interact with BMI categories and time. Based on the environmental constraints of nursing homes, we hypothesize that institutionalization will be a stronger determinant of functional performance than BMI, and that the trajectory of change over time (responsiveness to the protocol) will differ significantly between settings, overshadowing the expected limitations of obesity.

## 2. Materials and Methods

### 2.1. Participants

The sample size calculation was performed a priori using G*Power software (version 3.1.9.7; Heinrich-Heine-Universität Düsseldorf, Germany, Düsseldorf). The study design followed a 3 × 2 × 2 mixed factorial arrangement, comprising three BMI categories (Normal weight, Overweight, Obesity) and two living settings (Community-dwelling vs. Institutionalized) across two time points (Baseline vs. Post-16 weeks). The calculation was based on a repeated measures ANOVA design (within-between interaction), considering a significance level (alpha) of 0.05, a statistical power (1-beta) of 0.80, and a medium-to-large effect size (f = 0.25), and a conservative correlation among repeated measures of 0.50, derived from previous literature on functional decline in similar populations [10,36]. The analysis indicated that a minimum total sample size of 90 participants would be required to detect significant interactions. Given that our final sample consisted of 124 participants, the study is adequately powered to test the proposed hypotheses.

Participants were recruited from both community-dwelling and nursing home settings using a convenience sampling method facilitated by institutional staff. These two recruitment sources were treated as distinct cohorts, representing the typical demographic characteristics of their respective environments. To be eligible for the study, participants were required to meet the following inclusion criteria: (i) be aged 65 years or older; (ii) participate voluntarily and provide written informed consent; (iii) possess sufficient cognitive and sensory acuity (i.e., auditory and visual capabilities) to comprehend and execute testing protocols; (iv) exhibit independent ambulation without reliance on assistive devices (e.g., canes or walkers); and (v) have medical clearance to perform physical fall risk assessments. Conversely, participants were excluded if they had a known diagnosis of a cognitive disorder or neurological condition that could compromise physical functioning, mobility, or balance. Additionally, individuals with a history of recurrent falls, defined as experiencing two or more falls within the preceding six months, were not eligible for participation.

A total of 124 older adults volunteered to participate in this study. The detailed characteristics of the total sample and subgroups stratified by living setting are presented in Table 1. The sample was divided into two groups. Institutionalized (*n* = 65, 52.4%) and Community-dwelling (*n* = 59, 47.6%). As shown in Table 1, the institutionalized group was markedly older (mean age: 84.37 years) compared to the community-dwelling group (mean age: 70.31 years). Regarding body composition, the prevalence of obesity was twofold higher in the nursing home setting (33.8%) compared to the community (16.9%), whereas the community group presented a higher proportion of normal-weight individuals.

### 2.2. Procedures

The study protocol was reviewed and approved by the Ethics Committee of the Polytechnic of Leiria (protocol code CE/IPLEIRIA/63/2024, approved on 24 May 2024) and was conducted in strict adherence to the ethical standards of the Declaration of Helsinki for research involving human subjects. Data collection was performed using a convenience sampling method across two distinct settings: nursing homes and community-based physical activity programs. Specifically, the community-dwelling participants were recruited from established active aging cohorts, representing a physically active and high-functioning subgroup. The recruitment process followed a hierarchical authorization protocol. First, researchers contacted the clinical directors of the nursing homes and the coordinators of the community programs to explain the study objectives and obtain formal authorization to access the facilities and potential participants. Following institutional approval, clinical directors and program coordinators conducted an initial pre-screening of their residents/users based on the study’s inclusion criteria. Staff members then identified the pool of eligible individuals with the research team. Due to this partner-mediated recruitment process, the total number of individuals initially screened but excluded (e.g., due to medical ineligibility assessed internally by staff) was not recorded. The eligible participants identified were then approached individually in a private setting to ensure confidentiality. They received a detailed explanation of the objectives and procedures and were explicitly informed that participation was voluntary, anonymous, and independent of their standing in their respective institutions or programs. Participants had the opportunity to ask questions before providing written informed consent.

Upon inclusion, the assessment protocol began with the measurement of anthropometric variables (height and weight) to calculate BMI. Height was measured to the nearest 0.1 cm using a portable stadiometer (Seca 213, Hamburg, Germany), with participants standing upright, head in the Frankfurt horizontal plane. Body weight was measured to the nearest 0.1 kg using a portable digital scale (Seca 813, Hamburg, Germany), with participants wearing light clothing and no shoes. Subsequently, the 8-foot Up and Go test was administered, following the protocol of the Senior Fitness Test [9]. Before the test, researchers provided a clear demonstration and verbal instructions. Participants were instructed to rise from a chair, walk 2.44 m, turn around, walk back, and sit down. To ensure full comprehension and safety, participants performed one practice trial. Following the practice, participants completed two experimental trials with the instruction to perform the task as fast and safely as possible. The time was recorded using a stopwatch to the nearest 0.01 s, and only the best time (lowest duration) of the two trials was used for analysis. This entire assessment protocol was repeated after a 16-week period under the same conditions (T2). During this interval, participants were instructed to maintain their usual levels of physical activity and dietary habits to minimize confounding variables and ensure that changes in performance were not attributed to external lifestyle modifications.

### 2.3. Statistical Analysis

Descriptive statistics are presented as means ± standard deviations, along with medians and interquartile ranges to account for non-normal distribution patterns. Normality of the data was verified using the Shapiro–Wilk test, and homogeneity of variances was assessed using Levene’s test. A 3 × 2 × 2 repeated-measures GLM using the multivariate approach (Pillai’s Trace) was conducted to analyze the effects of Time (Baseline vs. 16 Weeks) as the within-subjects factor, and BMI Category (Normal vs. Overweight vs. Obese) and Living Setting (Community vs. Institution) as between-subjects factors. Given the violation of homogeneity of variances and covariance matrices, the Multivariate approach was adopted to assess within-subject effects and interactions. Specifically, Pillai’s Trace was used as the test statistic, as this approach is robust against violations of statistical assumptions in complex designs. When significant F-values were found, Bonferroni post hoc tests with 95% Confidence Intervals (95% CI) were used to identify specific differences between groups. To control for the significant age difference between groups, a complementary Analysis of Covariance (ANCOVA) was performed using age as a covariate to verify if the effect of Living Setting on 8UG performance remained significant independent of biological aging. Effect sizes for the GLM were reported as partial eta squared (η^2^_p_). All statistical analyses were performed using IBM SPSS Statistics software, version 31.0 (IBM Corp., Armonk, NY, USA), with a significance level set at *p* < 0.05.

## 3. Results

Prior to the inferential analysis, the assumptions for the repeated-measures GLM were assessed. The Shapiro–Wilk test indicated that the data for the 8UG test were not normally distributed within the subgroups (*p* < 0.05). Additionally, Levene’s test indicated unequal variances across groups (*p* < 0.001), and Box’s M test indicated a violation of the assumption of homogeneity of covariance matrices (M = 466.74, *p* < 0.001). Given these violations, particularly the heterogeneity of covariance matrices, we adopted the Multivariate approach for the repeated measures analysis. Consequently, Pillai’s Trace was used as the primary test statistic for determining the significance of within-subject effects and interactions, as it provides greater robustness to heteroscedasticity compared to the standard univariate F-test. The analysis indicated significant interaction effects (Pillai’s Trace = 0.196; F (1, 118) = 28.74; *p* < 0.001), allowing for interpretation of the results within this specific factorial design. Given the baseline age disparity between community-dwelling and institutionalized participants, an ANCOVA was conducted to determine if the differences in functional mobility were driven solely by biological aging. The results indicated that while age was a significant predictor of 8UG performance (F (1, 121) = 6.83, *p* = 0.010), the effect of living setting remained highly significant even after adjusting for age (F (1, 121) = 11.53, *p* < 0.001). This indicates that the association remained significant after adjustment for age. However, residual confounding cannot be excluded due to the strong baseline imbalance (see Table 2 for details).

It is important to highlight the high dispersion observed in the institutionalized normal-weight group (i.e., standard deviation ± 27.26 s at baseline). This variability indicates that the mean performance of this subgroup is skewed by specific outliers exhibiting severe functional dependence, rather than representing a homogenous characteristic of all normal-weight residents. Therefore, descriptive values for this specific subgroup should be interpreted with caution.

The analysis revealed a significant main effect for Time (F (1, 118) = 29.39, *p* < 0.001, η^2^_p_ = 0.199), confirming the overall reduction in 8UG completion time across the sample. Crucially, a significant main effect was found for Living Setting (F (1, 118) = 68.46, *p* < 0.001, η^2^_p_ = 0.367), reinforcing that community-dwelling older adults consistently outperformed their institutionalized peers. In contrast, BMI categories did not significantly influence 8UG performance (F (2, 118) = 0.90, *p* = 0.408, η^2^_p_ = 0.015), suggesting that weight status alone was not a distinctive factor in this context. The significant Time × Living Setting interaction (F (1, 118) = 28.74, *p* < 0.001, η^2^_p_ = 0.196) highlights that functional changes over the 16 weeks were setting dependent. No other interactions were significant (see Table 3 for complete details).

To further elucidate the Time × Living Setting interaction, an analysis of the marginal means was performed. The data revealed distinct trajectories. The Institutionalized group showed a significant time reduction, lowering their average time from 24.23 s at baseline to 20.33 s after 16 weeks (a reduction of ~16%). In contrast, the Community-dwelling group maintained a stable performance (T1: 4.68 s vs. T2: 4.69 s), likely due to a ceiling effect, as their baseline values were already indicative of high functional mobility. Table 4 presents the decomposition of the interaction using estimated marginal means. Pairwise comparisons suggest that the 16-week time period elicited a significant test–retest change in the institutionalized group (*p* < 0.001), showing a reduction of approximately 3.5 s in 8UG performance. In contrast, no significant changes were observed in community-dwelling older adults (*p* = 1.000), who maintained their high baseline functional levels throughout the 16 weeks.

## 4. Discussion

The primary aim of this study was to analyze the combined effects of BMI and living setting on 8UG performance over a 16-week period. Results indicated that the living setting (institutionalization vs. community) is a far stronger predictor of functional mobility than BMI-defined weight status, confirming our hypothesis regarding environmental dominance. Furthermore, the temporal response was highly context-dependent: institutionalized older adults showed significant reductions in test duration (i.e., responsiveness), whereas community-dwelling peers maintained their baseline levels.

Our results showed a marked baseline disparity (~24 s vs. ~4.6 s), aligning with the environmental docility hypothesis [41], where declining competence increases dependence on environmental pressures [17]. Consistently, literature suggests that while community-dwelling older adults preserve function through daily instrumental activities [18], the nursing home environment often promotes a confined, sedentary routine that accelerates deconditioning [8,41,42]. The magnitude of this effect (η^2^_p_ = 0.367) indicates that the profile associated with the living setting is strongly associated with functional performance. However, it is crucial to interpret this living setting effect as a composite proxy that encompasses not only the physical environment but also the participants’ advanced age, higher frailty levels, and care dependency. The institutionalized cohort was significantly older than the community group, and this specific combination of biological and environmental characteristics appears to diminish the relevance of BMI categories as a predictor, contrasting with the patterns observed in younger, community-dwelling cohorts [43,44]. A critical finding was the lack of a significant main effect for BMI (*p* = 0.408). Although the obesity paradox suggests protective effects of excess weight in geriatrics [31,33], our findings indicate that institutional constraints (e.g., sedentarism) impact mobility uniformly across weight categories. The institutional environment appears to mitigate the influence of individual anthropometric variations, minimizing the relevance of potential biological reserves or body mass advantages.

The Community-dwelling group maintained a stable performance (T1: 4.68 s vs. T2: 4.69 s). This stability is attributable to a pronounced ceiling effect. The baseline performance of this group (~4.6 s) aligns with the normative standards for the 8-foot Up-and-Go test established by Rikli and Jones [9], confirming their status as a “high functioning” subgroup, likely due to their recruitment from active aging programs. Their physiological margin for improvement was minimal, limiting the test’s sensitivity to detect further changes [11]. This extreme disparity creates a “functional floor”, where the potential for relative change is much higher compared to the stable, high-functioning community peers. Indeed, the institutionalized group showed a marked change in performance over the 16-week period, reducing their time from ~24 s to ~20 s. However, given the observational nature of the study where no structured exercise intervention was applied, these results should not be interpreted as physiological gains. Instead, the reduction in time is likely attributable to a learning effect and familiarization with the testing protocol, which tends to be more pronounced in individuals with lower initial functional capacity. This carries a critical clinical implication: baseline assessments in nursing homes may significantly overestimate fall risk due to the participants’ unfamiliarity with testing demands. The initial ‘slow’ performance may have reflected caution or cognitive uncertainty rather than purely physical incapacity. Therefore, a single 8UG trial may not reflect true motor capacity as familiarization sessions are essential to avoid false positives for high fall risk [5,45]. This indicates that these individuals retain functional capacity and latent motor reserve that can be activated even by minimal stimulation (i.e., reassessment), contradicting the ageist view that interventions in nursing homes may be ineffective [46,47].

The absence of a significant interaction between Time and BMI (*p* = 0.246) suggests that the trajectory of functional change is relatively uniform across weight categories when environmental factors are controlled. This reinforces the findings of recent meta-analyses suggesting that physical activity induces beneficial adaptations in gait and balance irrespective of baseline BMI [35,48]. However, the potential vulnerability of the normal weight institutionalized group warrants attention, although these specific findings should be considered exploratory and hypothesis-generating given the small sample size. Studies on sarcopenic obesity often highlight the double burden of fat and low muscle [32,49]. However, our data point towards a potential vulnerability in the normal-weight institutionalized subgroup. While we lacked direct measures to confirm muscle depletion, the poor performance of this group raises the hypothesis that phenotypes resembling sarcopenic thinness (i.e., low muscle mass masked by normal weight) might be a critical unaddressed risk factor in nursing homes [50]. However, these null findings regarding BMI should be interpreted with caution. While BMI did not differentiate fall risk in this sample, this does not rule out the critical role of muscle tissue quality. BMI is a metric that fails to distinguish between lean mass and adipose tissue. It is plausible that alternative metrics, such as appendicular lean mass or muscle quality (e.g., dynapenia), would have yielded different results. Previous literature suggests that muscle strength is a more potent mediator of fall risk than body mass per se [36,47]. For instance, an individual with normal weight by BMI standards may suffer from sarcopenia, hiding a potential fall risk, whereas an individual with obesity might possess sufficient muscle strength to compensate for excess weight. Therefore, future assessments should prioritize functional measures of strength over anthropometric weight status alone.

Some limitations must be acknowledged to contextualize these findings. First, the sample size for the “Normal Weight” institutionalized group was small (*n* = 9). This limitation is compounded by the extreme heterogeneity observed in this subgroup (SD ± 27.26 s), suggesting that mean values were likely skewed by outliers exhibiting severe functional impairment. Consequently, the apparent poor performance of this phenotype should be interpreted as a conditional trend heavily influenced by specific cases of advanced frailty, rather than a uniform characteristic. Second, the community-dwelling sample was drawn from active aging programs and presents functional values superior to those of the general population. Therefore, results for this group should be generalized to active older adults rather than the broader, potentially sedentary, community population. Third, we used BMI as a proxy for body composition; however, BMI cannot distinguish between fat mass and muscle mass, nor does it account for muscle strength. Consequently, we could not definitively classify participants as sarcopenic or sarcopenic-obese, making our interpretations regarding muscle quality speculative. Future studies should employ bioimpedance or DEXA to directly assess muscle mass, alongside simple field metrics (e.g., handgrip strength or calf circumference), to vigorously test the sarcopenia hypothesis [3]. Fourth, the study design did not control for the specific type of medication (e.g., psychotropics), which is known to influence balance and is highly prevalent in nursing homes [51]. Fifth, although participants were instructed to maintain their usual dietary habits and physical activity levels, these variables were not strictly monitored via logs or accelerometry. However, since no new structured exercise or rehabilitation program was introduced during this period, it is plausible to assume that the changes in 8UG performance are predominantly driven by the learning effect described. As this was a longitudinal observational study, we cannot rule out that unmeasured variations in daily activity might have influenced the functional trajectories. Finally, given the multicenter nature of the data collection, there is potential heterogeneity regarding the specific characteristics of different nursing homes and community programs (e.g., quality of care or facilities). While this increases ecological validity, we could not control for a potential center effect. Future research should investigate whether multimodal exercise interventions can bridge the gap between institutionalized and community-dwelling older adults, specifically targeting muscle power in normal-weight institutionalized residents, who appear to represent the most vulnerable phenotype [52].

## 5. Conclusions

This study suggests that the profile associated with the living setting, including age-related frailty and environmental constraints, is a critical factor associated with functional mobility more than BMI measured in categories. Our results may challenge the utility of BMI as a standalone predictor of fall risk in institutionalized settings, as the characteristics of the institutionalized cohort appeared to mitigate the expected variances across BMI categories. Consequently, fall prevention strategies should prioritize cognitive and motor familiarization before diagnosis, preventing the overestimation of physical decline.

## Figures and Tables

**Table 1:** Baseline characteristics of the participants are stratified by living setting.

Characteristics	Total Sample (*n* = 124)	Community-Dwelling (*n* = 59)	Institutionalized (*n* = 65)
Age (years), Mean ± SD	77.68 ± 9.31	70.31 ± 3.61	84.37 ± 7.69
Sex, *n* (%)			
Male	37 (29.8%)	19 (32.2%)	18 (27.7%)
Female	87 (70.2%)	40 (67.8%)	47 (72.3%)
BMI Category, *n* (%)			
Normal weight	25 (20.2%)	16 (27.1%)	9 (13.8%)
Overweight	67 (54.0%)	33 (55.9%)	34 (52.3%)
Obesity	32 (25.8%)	10 (16.9%)	22 (33.8%)

Notes: Data are presented as mean ± standard deviation (SD) for continuous variables and frequency (percentage) for categorical variables. BMI: Body Mass Index.

**Table 2:** Descriptive statistics for the 8UG test performance (seconds) at baseline (T1) and post-16 weeks (T2), stratified by living setting and BMI.

Living Setting	BMI Category	*n*	8UG1 (Baseline)				8UG2 (Post-16 Weeks)			
			M	SD	Mdn	IQR	M	SD	Mdn	IQR
Institutionalized	Normal weight	9	28.84	27.26	16.56	41.61	27.02	24.90	17.33	36.90
	Overweight	34	22.55	14.50	17.95	17.58	18.86	13.09	13.32	12.38
	Obese	22	24.93	12.21	22.08	16.87	19.87	12.23	16.58	16.30
	Total	65	24.23	15.97	20.02	17.85	20.33	14.91	14.43	14.47
Community	Normal weight	16	4.65	0.55	4.64	1.00	4.48	0.58	4.46	0.88
	Overweight	33	4.54	0.78	4.19	0.70	4.62	0.85	4.44	1.08
	Obese	10	5.20	0.95	5.08	1.47	5.23	0.96	5.03	1.57
	Total	59	4.68	0.78	4.32	1.14	4.69	0.83	4.51	1.16
Total Sample		124	14.93	15.13	8.13	16.80	12.89	13.33	7.82	10.18

Notes: M = Mean; SD = Standard Deviation; IQR = Interquartile Range.

**Table 3:** Summary of the Mixed ANOVA results examining the effects of Time, Living Setting, and BMI on Timed Up and Go performance.

Model	df	F	Sig.	η^2^_p_
Between-Subjects Effects				
Living Setting	1	68.46	<0.001	0.367
BMI Category	2	0.90	0.408	0.015
Living Setting × BMI	2	0.92	0.401	0.015
Within-Subjects Effects				
Time	1	29.39	<0.001	0.199
Time × Living Setting	1	28.74	<0.001	0.196
Time × BMI	2	1.42	0.246	0.024
Time × Living Setting × BMI	2	1.84	0.163	0.030

Note: df = degrees of freedom; F = F-statistic; η^2^_p_ = partial eta squared.

**Table 4:** Bonferroni-adjusted pairwise comparisons of 8UG performance (Estimated Marginal Means) between time points within each Living Setting.

Living Setting	Comparison	Mean Difference (T1–T2)	Std. Error	*p*-Value	95% CI
Institutionalized	Baseline vs. Post-16 Weeks	3.52	0.65	<0.001 *	[2.23, 4.81]
Community	Baseline vs. Post-16 Weeks	0.02	0.66	1.000	[−1.29, 1.33]

Note: Mean Diff. = Mean Difference (seconds); CI = Confidence Interval for Difference. * Significant differences (*p* < 0.05). Comparisons are adjusted using the Bonferroni correction.

## Data Availability

The data presented in this study is available on request from the corresponding author. The data is not publicly available due to privacy and ethical restrictions regarding participant anonymity.

## References

[1] Beauchet O., Fantino B., Allali G., Muir S.W., Montero-Odasso M., Annweiler C. (2011). Timed Up and Go test and risk of falls in older adults: A systematic review. J. Nutr. Health Aging.

[2] Milanović Z., Pantelić S., Trajković N., Sporiš G., Kostić R., James N. (2013). Age-related decrease in physical activity and functional fitness among elderly men and women. Clin. Interv. Aging.

[3] Cruz-Jentoft A.J., Bahat G., Bauer J., Boirie Y., Bruyère O., Cederholm T., Cooper C., Landi F., Rolland Y., Sayer A.A. (2019). Sarcopenia: Revised European consensus on definition and diagnosis. Age Ageing.

[4] Ryu M., Jo J., Lee Y., Chung Y.-S., Kim K.-M., Baek W.-C. (2013). Association of Physical Activity with Sarcopenia and Sarcopenic Obesity in Community-Dwelling Older Adults: The Fourth Korea National Health and Nutrition Examination Survey. Age Ageing.

[5] Podsiadlo D., Richardson S. (1991). The Timed “Up & Go”: A Test of Basic Functional Mobility for Frail Elderly Persons. J. Am. Geriatr. Soc..

[6] Benavent-Caballer V., Sendín-Magdalena A., Lisón J.F., Rosado-Calatayud P., Amer-Cuenca J.J., Salvador-Coloma P., Segura-Ortí E. (2016). Physical factors underlying the Timed “Up and Go” test in older adults. Geriatr. Nurs..

[7] Nightingale C.J., Mitchell S.N., Butterfield S.A. (2019). Validation of the Timed Up and Go Test for Assessing Balance Variables in Adults Aged 65 and Older. J. Aging Phys. Act..

[8] Rodrigues F., Teixeira J.E., Forte P. (2023). The Reliability of the Timed Up and Go Test among Portuguese Elderly. Healthcare.

[9] Rikli R.E., Jones C.J. (2013). Development and Validation of Criterion-Referenced Clinically Relevant Fitness Standards for Maintaining Physical Independence in Later Years. Gerontologist.

[10] Bohannon R.W. (2006). Reference Values for the Timed Up and Go Test: A Descriptive Meta-Analysis. J. Geriatr. Phys. Ther..

[11] Mayhew A.J., So H.Y., Ma J., Beauchamp M.K., Griffith L.E., Kuspinar A., Lang J.J., Raina P. (2023). Normative values for grip strength, gait speed, timed up and go, single leg balance, and chair rise derived from the Canadian longitudinal study on ageing. Age Ageing.

[12] Gusi N., Prieto J., Olivares P.R., Delgado S., Quesada F., Cebrián C. (2012). Normative Fitness Performance Scores of Community-Dwelling Older Adults in Spain. J. Aging Phys. Act..

[13] Chandhanayingyong C., Adulkasem N., Asavamongkolkul A., Chotiyarnwong P., Vanitcharoenkul E., Laohaprasitiporn P., Soparat K., Unnanuntana A. (2024). Establishing Normative Values for Performance-Based Tests in Older Thai Adults: A Nationwide Cross-Sectional Study. Arch. Phys. Med. Rehabil..

[14] Chung P.-K., Zhao Y., Liu J.-D., Quach B. (2016). Functional fitness norms for community-dwelling older adults in Hong Kong. Arch. Gerontol. Geriatr..

[15] Wang C.-W., Yeh J.-L., Li S.-F., Chen C.-M., Wang H.-H., He C.-S., Lin H.-T. (2024). Functional Fitness Norms of Community-Dwelling Older Adults in Southern Rural Taiwan: A Cross-Sectional Study. Healthcare.

[16] Ibrahim A., Singh D.K.A., Shahar S. (2017). ‘Timed Up and Go’ test: Age, gender and cognitive impairment stratified normative values of older adults. PLoS ONE.

[17] Tariq A., Zadeh S.A.M., Ammar M., Mousavizadeh N., Hajary A., Mohamadi S. (2025). Relationship between multiple morbidities and performance on the Timed Up and Go test in elderly patients: A cross-sectional study. BMJ Open.

[18] Cossio-Bolaños M., Vidal-Espinoza R., Villar-Cifuentes I., de Campos L.F.C.C., de Lázari M.S.R., Urra-Albornoz C., Sulla-Torres J., Gomez-Campos R. (2024). Functional fitness benchmark values for older adults: A systematic review. Front. Public Health.

[19] Cossio-Bolaños M., Vidal-Espinoza R., Castelli Correia de Campos L.F., Sulla-Torres J., Urra-Albornoz C., Alvear-Vasquez F., Fuentes-Lopez J., Ramos de Lazari M.S., Gomez-Campos R. (2025). Normative values to assess functional fitness in older adults in a region of Chile. Front. Aging.

[20] Gouveia É.R., Maia J.A., Beunen G.P., Blimkie C.J., Fena E.M., Freitas D.L. (2013). Functional Fitness and Physical Activity of Portuguese Community-Residing Older Adults. J. Aging Phys. Act..

[21] Marques E.A., Baptista F., Santos R., Vale S., Santos D.A., Silva A.M., Mota J., Sardinha L.B. (2014). Normative Functional Fitness Standards and Trends of Portuguese Older Adults: Cross-Cultural Comparisons. J. Aging Phys. Act..

[22] Magalhães J.P., Hetherington-Rauth M., Rosa G.B., Correia I.R., Pinto G.M., Ferreira J.P., Coelho-e-Silva M.J., Raimundo A.M., Mota J., Sardinha L.B. (2023). Functional fitness trends among older adults in Portugal between 2008 and 2018: Keeping up with a healthy aging process. J. Sci. Med. Sport.

[23] Rodrigues F., Monteiro D., Matos R., Jacinto M., Antunes R., Gomes P., Amaro N. (2024). Physical Fitness of the Older Adult Community Living in Leiria, Portugal. Epidemiologia.

[24] Lima A.B., Faber M.A., Peralta M., Vila-Suárez H., Henriques-Neto D. (2025). Functional Fitness of Low-Income Community-Dwelling Older Adults in Amazonian Brazilian. Healthcare.

[25] Ascencio E.J., Cieza-Gómez G.D., Carrillo-Larco R.M., Ortiz P.J. (2022). Timed up and go test predicts mortality in older adults in Peru: A population-based cohort study. BMC Geriatr..

[26] Gatenio-Hefling O., Tzemah-Shahar R., Asraf K., Dilian O., Gil E., Agmon M. (2025). Revisiting the “Timed Up and Go” test: A 12-s cut-off can predict Hospitalization Associated Functional Decline in older adults. GeroScience.

[27] Tóth E.E., Vujić A., Ihász F., Ruíz-Barquín R., Szabo A. (2025). Functional fitness and psychological well-being in older adults. BMC Geriatr..

[28] Bohrer R.C.D., Bianchi A.S., Balcetis E., Wong B.W.C., Rodacki A.L.F. (2025). Perceived self-efficacy and body mass index to identify older adults with and without fall history. Obes. Res. Clin. Pract..

[29] Asai T., Oshima K., Fukumoto Y., Yonezawa Y., Matsuo A., Misu S. (2018). Association of Fall History with the Timed Up and Go test score and the dual task cost: A cross-sectional study among independent community-dwelling older adults. Geriatr. Gerontol. Int..

[30] Coelho-Junior H.J., Rodrigues B., de Oliveira Gonçalves I., Asano R.Y., Uchida M.C., Marzetti E. (2018). The physical capabilities underlying timed “Up and Go” test are time-dependent in community-dwelling older women. Exp. Gerontol..

[31] Ling C., Kelechi T., Mueller M., Brotherton S., Smith S. (2012). Gait and Function in Class III Obesity. J. Obes..

[32] Ferhi H., Maktouf W. (2023). The impact of obesity on static and proactive balance and gait patterns in sarcopenic older adults: An analytical cross-sectional investigation. PeerJ.

[33] Neto I.V.S., Diniz J.S., Alves V.P., Oliveira A.R.V., Barbosa M.P.S., Prado C.R.S., Alencar J.A., Silva K.H.C.V., Silva C.R., Ferreira G.M.L. (2023). Field-Based Estimates of Muscle Quality Index Determine Timed-Up-and-Go Test Performance in Obese Older Women. Clin. Interv. Aging.

[34] Shim G.Y., Yoo M.C., Soh Y., Chon J., Won C.W. (2024). Obesity, Physical Performance, Balance Confidence, and Falls in Community-Dwelling Older Adults: Results from the Korean Frailty and Aging Cohort Study. Nutrients.

[35] Tavares M.S., Menezes S.L.S., Ribeiro E.D.F., Orsini M., Tuza F.A., de Moura P.H., Terra D.V., Moreno A.M. (2024). Associations between Cardiovascular Risk Factors and Timed Up and Go Test for Elderly Participants in Public Physical Activity Programs. Int. J. Environ. Res. Public Health.

[36] Rodrigues F., Izquierdo M., Monteiro D., Jacinto M., Matos R., Amaro N., Antunes R., Teixeira D.S. (2024). Muscle Strength Matters Most for Risk of Falling Apart from Body Mass Index in Older Adults: A Mediated-Moderation Analysis. J. Frailty Aging.

[37] Tuna H.D., Edeer A.O., Malkoc M., Aksakoglu G. (2009). Effect of age and physical activity level on functional fitness in older adults. Eur. Rev. Aging Phys. Act..

[38] Adamo D.E., Talley S.A., Goldberg A. (2015). Age and Task Differences in Functional Fitness in Older Women: Comparisons With Senior Fitness Test Normative and Criterion-referenced Data. J. Aging Phys. Act..

[39] Sardinha L.B., Santos D.A., Marques E.A., Mota J. (2015). Criterion-referenced fitness standards for predicting physical independence into later life. Exp. Gerontol..

[40] Zhao Y., Chen P., Gong Y., Gu Z., Zhou W. (2024). Functional fitness and physical independence in later years: Cut-off values and validation. BMC Geriatr..

[41] Lawton M.P., Simon B. (1968). The ecology of social relationships in housing for the elderly. Gerontologist.

[42] Theou O., Blodgett J.M., Godin J., Rockwood K. (2017). Association between sedentary time and mortality across levels of frailty. CMAJ.

[43] Kojima G. (2015). Prevalence of frailty in nursing homes: A systematic review and meta-analysis. J. Am. Med. Dir. Assoc..

[44] Clegg A., Young J., Iliffe S., Rikkert M.O., Rockwood K. (2013). Frailty in elderly people. Lancet.

[45] Perera S., Mody S.H., Woodman R.C., Studenski S.A. (2006). Meaningful change and responsiveness in common physical performance measures in older adults. J. Am. Geriatr. Soc..

[46] Fiatarone Singh M.A. (2004). Exercise in the oldest old. J. Am. Geriatr. Soc..

[47] Valenzuela T. (2012). Efficacy of progressive resistance training interventions in older adults in nursing homes: A systematic review. J. Am. Med. Dir. Assoc..

[48] Villareal D.T., Chode S., Parimi N., Sinacore D.R., Hilton T., Armamento-Villareal R., Napoli N., Qualls C., Shah K. (2011). Weight loss, exercise, or both and physical function in obese older adults. N. Engl. J. Med..

[49] Stenholm S., Harris T.B., Rantanen T., Visser M., Kritchevsky S.B., Ferrucci L. (2008). Sarcopenic obesity: Definition, cause and consequences. Curr. Opin. Clin. Nutr. Metab. Care.

[50] Landi F., Cruz-Jentoft A.J., Liperoti R., Russo A., Giovannini S., Tosato M., Capoluongo E., Bernabei R., Onder G. (2013). Sarcopenia and mortality risk in frail older persons aged 80 years and older: Results from ilSIRENTE study. Age Ageing.

[51] Woolcott J.C., Richardson K.J., Wiens M.O., Patel B., Marin J., Khan K.M., Marra C.A. (2009). Meta-analysis of the impact of 9 medication classes on falls in elderly persons. Arch. Intern. Med..

[52] Cadore E.L., Rodríguez-Mañas L., Sinclair A., Izquierdo M. (2013). Effects of different exercise interventions on risk of falls, gait ability, and balance in physically frail older adults: A systematic review. Rejuvenation Res..

